# Results of a patient survey for an implantable neurostimulator to treat migraine headaches

**DOI:** 10.1007/s10194-012-0430-0

**Published:** 2012-03-07

**Authors:** Koen Paemeleire, Amy M. Goodman

**Affiliations:** 1 Department of Neurology, Ghent University Hospital, De Pintelaan 185, 9000 Ghent, Belgium; 2 Clinical Affairs, Autonomic Technologies, Inc, 3698 Haven Avenue, Suite C, Redwood City, CA 94063 USA

**Keywords:** Sphenopalatine ganglion (SPG), Migraine, Headache, Neuromodulation, Neurostimulation

## Abstract

Migraine attacks are believed to involve activation of the trigeminovascular system and trigeminal-parasympathetic reflex, which is mediated through the sphenopalatine ganglion (SPG). An implantable SPG neurostimulator has been developed to apply on-demand SPG stimulation for the treatment of severe primary headache. The neurostimulator is implanted via an oral incision and placed along the maxilla, with the lead placed at the SPG. The neurostimulator contains no battery and is powered and controlled via a handheld remote controller. The potential interest of patients with high-frequency, high-disability migraine in having a SPG neurostimulator implanted to treat migraine is unknown. We aimed to evaluate patient interest to undergo such an implantation procedure and to participate in a clinical investigation of on-demand SPG stimulation for migraine by conducting a survey at the Ghent University Hospital in 41 migraineurs. Seventy-seven percent (77%) of subjects expressed an interest in participating in a clinical investigation requiring implantation of a SPG neurostimulator when headache frequency and severity were considered and 69% when pain relief experienced with current migraine treatment was considered. Preventive and acute medications were used in 64 and 95% of the subjects, respectively, and provided a reported reduction of headache frequency, duration and pain. However, acute medications were frequently associated with headache recurrence and bothersome side effects. Results indicate that a majority of high-frequency, high-disability migraineurs, many of whom achieve pain relief with their current medications, have an interest in participating in a clinical investigation of an implantable SPG neurostimulator for the treatment of migraine headache.

## Introduction

The sphenopalatine ganglion (SPG) is an extracranial parasympathetic neural structure located in the pterygopalatine fossa. Migraine pain is believed to result from activation of the trigeminovascular system along with a trigeminal-parasympathetic reflex arc, which is mediated through the SPG [1, 2]. Cranial autonomic symptoms, which can be unilateral, are often associated with migraine headaches. These features are present in between 30 and 70% of migraine patients and include conjunctival injection, lacrimation, nasal congestion, and rhinorrhea [3]. These symptoms are the result of activation of the cranial parasympathetic system, and are believed to be due to activation of the trigeminal afferent arm of the trigeminal-parasympathetic reflex [4].

SPG interventions have been used for over 100 years to treat headache pain [5]. These SPG procedures include pharmacological blocks [6], lesional and non-lesional ablations [7] and surgery [8]. Despite purposefully damaging or destroying the SPG as part of the therapy, results have been good with minimal side effects, although the procedures have not provided permanent headache relief. Therefore, the potential of electrical stimulation of the SPG has been explored in pilot studies for acute treatment of cluster and migraine headaches using a temporary electrode [9, 10]. More recently, on-demand SPG stimulation for the treatment of chronic cluster headache has been evaluated in the Pathway CH-1 study using a miniaturized neurostimulator developed by Autonomic Technologies, Inc. (ATI). The neurostimulator is implanted through a gingival buccal incision using standard oral surgery techniques and placed along the maxilla with the lead located at the SPG within the pterygopalatine fossa. Following implantation, a “titration” period allows for refinement of stimulation settings. During the experimental period, headaches were randomized to one of three stimulation doses, including full-, sub-perception and placebo stimulation.

The potential interest of patients with high-frequency, high-disability migraine in undergoing implantation of a SPG neurostimulator to treat migraine headache is unknown. A multi-center clinical investigation, the Pathway M-1 trial, is underway in Europe to evaluate both the acute and preventive effects of on-demand SPG stimulation. We aimed to evaluate patient interest in undergoing such an implantation procedure and participation in a clinical investigation of on-demand SPG stimulation for migraine.

## Methods

A patient survey was conducted in 41 migraine patients at the Ghent University Hospital. Patients were required to have a MIDAS score of III to IV or a HIT-6 score greater than 56, to have 2–15 migraine pain days per month and to not have medication-overuse headache. Subjects were asked to consider their headache frequency and severity and their satisfaction with their current migraine treatment and to evaluate their willingness to undergo implantation of a neurostimulator for a clinical evaluation of SPG stimulation for migraine.

**Fig. 1:**
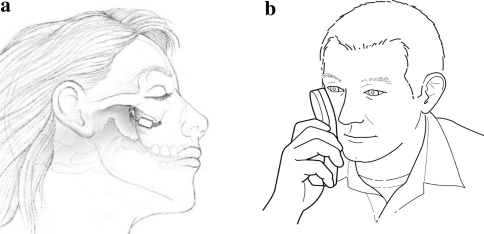
**a** Diagram showing the location of the implant behind the cheekbone. **b** Diagram showing how the remote controller is held over the cheek to control the activation of the neurostimulator

Patients were provided with general information regarding methods for using the ATI Neurostimulation System including that the neurostimulator is powered and controlled by holding a remote controller to the face (Fig. 1b). The implantation procedure was described as being similar to other types of oral surgery and requiring a few weeks to heal. Specific procedure-related adverse events were not provided. Specifically, patients were informed that the neurostimulator would be implanted through the mouth and placed behind the cheekbone (Fig. 1a), would not be visible after implant and would have no battery and thus would not require replacement, though if needed, could be removed using local anesthetic. The survey was designed to assess the percentage of attacks expected to be adequately treated in order for the patient to consider participation in a clinical investigation, thus, information regarding expectations for pain relief was not provided.

## Results

The subject population (Tables 1, 2) for the survey consisted largely of female high-frequency, high-disability episodic migraineurs with a high percentage of associated migraine symptoms. Subjects were recruited from the Outpatient Neurology Clinic of the Ghent University Hospital. Of the 41 subjects surveyed, 64% currently used preventive medication for their migraines, which were reported to reduce both migraine frequency (65%) and duration of the attacks (52%), but caused bothersome side effects in some subjects (24%). Thirty-nine subjects (95%) used acute medications which provided pain relief (75%) and pain freedom (68%), but were frequently associated with both headache recurrence (93%) and bothersome side effects (69%).

**Table 1:** Subject population

Gender	Female 79%
Age	37 (range 18–76)
Years of migraines	17 (range 2–66)
Frequency	Attacks/month: 7 (range 2–15)
	Days/month: 11 (range 4–20)
Duration	<1 day: 9%
	1–2 days: 55%
	2 days: 36%
Symptoms	Nausea/vomiting: 66%
	Light sensitivity: 90%
	Sound sensitivity: 76%
Disability scores	MIDAS
	I: 2 III: 13
	II: 0 IV: 26
	HIT-6
	64 (range 57–72)

**Table 2:** Preventive and acute medication usage

Preventive medication usage	Acute medication usage
64% Currently taking preventive medication	95% Currently taking acute medications
65% Reduction in migraine frequency	75% Pain reduction
52% Reduction in headache duration	68% Pain freedom
24% Bothersome side effects	93% Migraine headache recurrence
	69% Bothersome side effects

The majority of subjects used both preventive and acute medications. Although these medications were reported to reduce migraine frequency, duration and pain, the majority of subjects indicated they would consider participating in an investigation of a SPG neurostimulator for migraine. Specifically, given headache frequency/severity, and given the pain relief experienced with current migraine treatment, 77 and 69% of subjects, respectively, indicated a willingness to consider participating in a clinical investigation requiring implantation of a SPG neurostimulator. Of the subjects who would consider receiving an implant and participating in the study, 100% expected the therapy to treat at least 50%, and 81% expected the therapy to treat at least 75% of their migraines. Thus, the survey indicated an expectation that an implanted device to treat migraine should treat a majority of migraine attacks.

## Conclusion

Sphenopalatine ganglion stimulation using an implanted, on-demand neurostimulator is a novel, promising therapy option for migraine sufferers, although the ability of SPG stimulation to provide acute relief of migraine pain has not yet been demonstrated. Results of the survey conducted at the Ghent University Hospital indicate that a majority of high-frequency, high-disability migraineurs, many of whom achieve pain relief with their current medications, are willing to undergo implantation of a SPG neurostimulator and participate in a clinical investigation of SPG stimulation for the treatment of migraine headache, expecting a very efficacious non-pharmacological therapy alternative. Patient expectations regarding pain relief should be assessed and discussed prior to enrollment in the study. Detailed information on potential side effects and adverse events will further influence a patient’s decision to participate, but was not included in this survey.
